# Supplementary figures and images for: The contribution of Nintendo Wii Fit series in the field of health: a systematic review and meta-analysis
Source: PeerJ. 2017 Sep 5;5:e3600. doi: 10.7717/peerj.3600 (PMC5590553; doi:10.7717/peerj.3600)

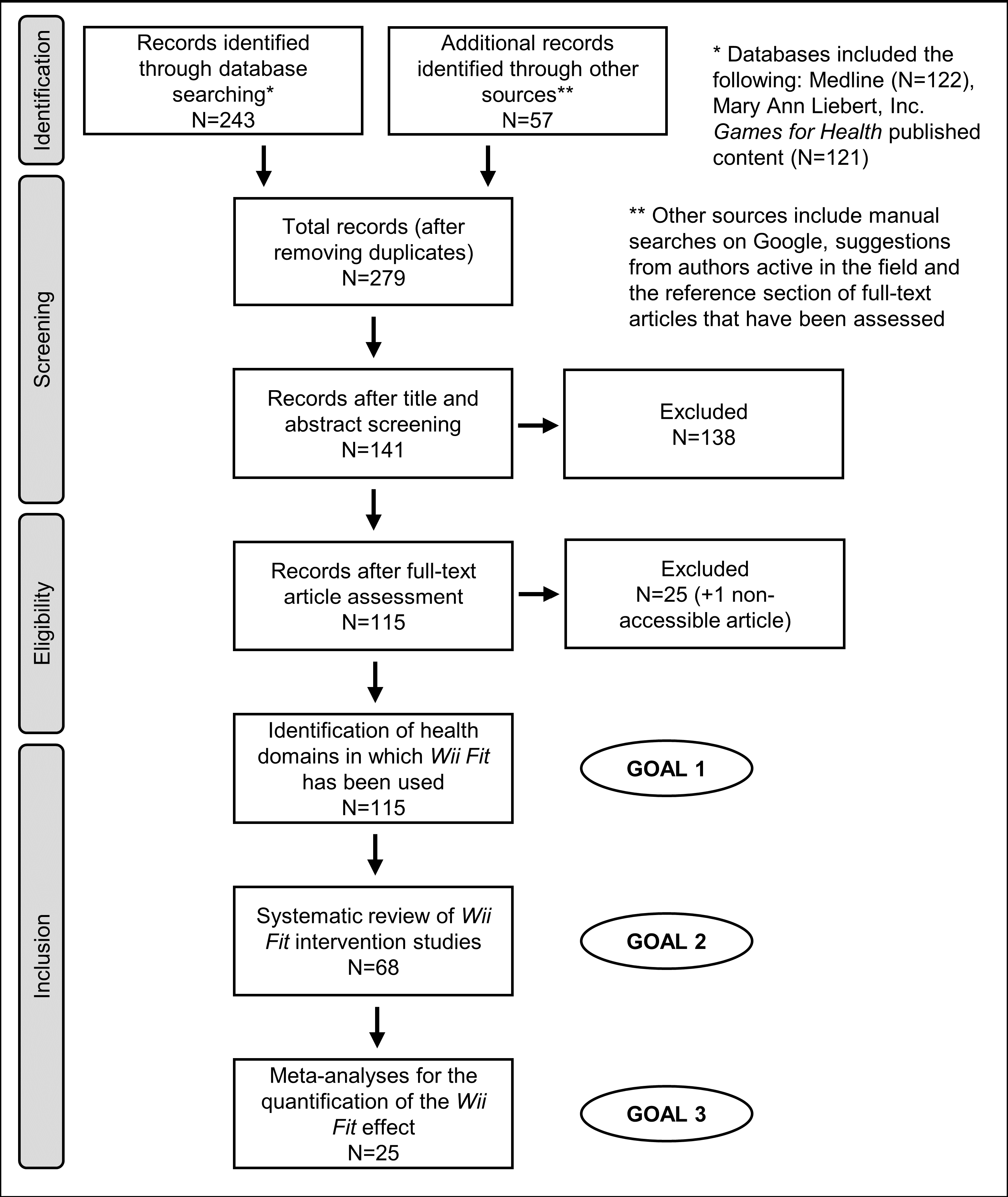

Supplement: Supplemental Information 1 [file peerj-05-3600-s001.png]
